# HSP90 and HSP70: Implication in Inflammation Processes and Therapeutic Approaches for Myeloproliferative Neoplasms

**DOI:** 10.1155/2015/970242

**Published:** 2015-10-15

**Authors:** Margaux Sevin, François Girodon, Carmen Garrido, Aurélie de Thonel

**Affiliations:** ^1^INSERM, UMR 866, Equipe Labellisée Ligue contre le Cancer and Association pour la Recherche contre le Cancer, La Ligue Nationale contre le Cancer, Laboratoire d'Excellence LipSTIC, 21000 Dijon, France; ^2^Faculty of Medicine and Pharmacy, University of Burgundy, 21000 Dijon, France; ^3^Service d'Hématologie Biologique, Pôle Biologie, 21000 Dijon, France; ^4^Centre Anticancéreux Georges-François Leclerc, CGFL, 21000 Dijon, France

## Abstract

Myeloproliferative neoplasms (MPN) are clonal stem cell disorders that lead to the excessive production of one or more blood cell lineages. It has been reported that, in most MPN, inflammatory cytokines are frequently increased, indicating that inflammation plays a crucial role in these disorders. Heat shock proteins (HSP) are induced in response to many stressful conditions from heat shock to hypoxia and inflammation. Besides their chaperone and cytoprotective functions, HSPs are key players during inflammation, hence the term “chaperokine.” Through their chaperone activity, HSP90, a stabilizer of many oncogenes (e.g., JAK2), and HSP70, a powerful antiapoptotic chaperone, tightly regulate Nuclear Factor-kappa B signalling, a critical pathway in mediating inflammatory responses. In light of this potential, several HSP90 inhibitors have been generated as anticancer agents able to degrade oncogenes. As it turns out, however, these drugs are also potent inhibitors of the inflammatory response in various diseases. Given the chaperone potential of HSP70 and the fact that HSP90 inhibitors induce HSP70, interest in HSP70 inhibitors is also increasing. Here, we focus on the implication of HSP90 and HSP70 in inflammatory responses and on the emergence of new therapeutic approaches in MPN based on HSP inhibitors.

## 1. Introduction 

### 1.1. Philadelphia Chromosome-Negative Myeloproliferative Neoplasms

Philadelphia chromosome-negative myeloproliferative neoplasms (MPNs) are acquired clonal disorders of haematopoietic stem cells (HSC) characterized by hyperplasia of one or several myeloid lineages. They include essential thrombocythaemia (ET), polycythaemia vera (PV), and myelofibrosis (PMF). The V617F mutation of the Janus kinase protein, JAK2, is the most prevalent genetic abnormality in these three types of MPN and is found in 95% of PV, and about 50% of ET and PMF [1–4]. This mutation, which usually affects only one of the JAK2 gene alleles in ET, frequently becomes homozygous in PV and MF. Subsequently, JAK2V617F induces the constitutive activation of downstream signalling pathways including PI3K (Phosphatidyl-Inositol-3 Kinase), MAPK (Mitogen Activating Protein Kinase), and STAT (Signal Transducers and Activators of Transcription) and thus cytokine independent growth and hypersensitivity [2]. Other abnormalities in the TPO receptor (MPL)/JAK2 axis, such as mutations in MPL, LNK, [5] or CBL epigenetic regulators (TET2 (Tet methylcytosine dioxygenase 2) [6] and DNMT3A (DNA Methyl Transferase 3b) [7]), have been identified. More recently, two groups have identified novel alterations of the calreticulin gene (CALR) in around 67% and 88% of JAK2-negative ET and PMF, respectively. These alterations were exceptionally found in PV patients. Inflammation seems to be independent from the identified mutations, and better understanding of the causes and molecular mechanisms that underlie chronic inflammation in MPNs seems necessary to improve the treatments currently proposed to MPN patients. Depending on the beneficial effects of JAK2 inhibitors on inflammatory conditions observed in myelofibrosis, one may reasonably wonder whether other anti-inflammatory therapeutics could be useful. In this review, we focus on the key role of heat shock proteins in inflammatory responses and on the emergence of new therapeutic approaches based on HSP inhibitors.

### 1.2. Heat Shock Proteins (HSPs)

Stress or heat shock proteins (HSPs), first discovered in 1962 by Ritossa [8], are a set of ubiquitous and highly conserved proteins. Mammalian HSPs have been classified into two groups according to their size: high molecular weight HSPs and small molecular weight HSPs. The first group includes four major families: HSP110, HSP90, HSP70, and HSP60. Some of these are expressed constitutively whereas expression of the others is induced by stressful conditions [9]. High molecular weight HSPs are ATP-dependent chaperones and require cochaperones to modulate their conformation and ATP binding. In contrast, small molecular weight HSPs, such as HSP27, are ATP-independent chaperones. HSPs are induced by a variety of physiological and environmental insults, from temperature stress to hypoxia, inflammation, infections, or anticancer chemotherapy [10]. Even in the absence of stress, HSPs play key roles in living systems by acting as chaperones. They assist in (i) the folding of newly synthesized polypeptides, (ii) the assembly of multiprotein complexes, and (iii) the transport of proteins across cellular membranes [11].

Stress proteins allow cells to survive in otherwise lethal conditions, and several mechanisms account for their cytoprotective effect: (i) as mentioned above, they are powerful chaperones; (ii) they participate in the proteasome-mediated degradation of proteins under stress conditions, thereby contributing to the so-called “protein triage”; (iii) they inhibit key effectors of the apoptotic machinery at the pre- and postmitochondrial level [12]. Among the different HSPs, HSP27 and HSP70 are the most strongly induced after stresses such as anticancer drugs, oxidative stress, radiation, and shock inflammatory stress. This need for HSPs increases not only after proteotoxic damage, but also during physiological conditions, such as differentiation processes, in a tissue and stage-specific manner. HSPs like HSP90 and HSP70 participate in the monomacrophagic differentiation of primary monocytes [13, 14]. In zebrafish, mutation of GRP75 (HSP70 family) specifically impairs the development of erythrocytes, granulocytes, and haematopoietic progenitors, thus giving rise to a human myelodysplastic-like syndrome (MDS) [15]. Moreover, HSP70 and HSP27 are required for erythroid differentiation of human primary erythroblasts [16, 17]. Apart from their cytoprotective functions, HSPs such as HSP90 and HSP70 have been shown to have additional cellular functions directly related to inflammation and the innate immune response. The term “chaperokine” was therefore attributed to these HSPs, which combine their unique function to act both as a chaperone and as cytokine (see Section 3).

In this review, we will focus mainly on the role of HSP90 and HSP70 in inflammation and on the therapeutic approaches based on their inhibition. 

*The Chaperone HSP90*. The HSP90 family includes HSP90alpha and HSP90beta, which functions as an ATP-dependent chaperone. HSP90 forms large protein complexes with other molecular chaperones (p23, cdc37, HSP70, and so on) known as multichaperone complexes. This chaperone complex is involved in the folding, activation, and assembly of numerous proteins, including key mediators of signal transduction and transcriptional regulation [18]. The alpha and beta isoforms of HSP90, which are essential for the viability of eukaryotic cells, represent 1-2% of total cytosolic proteins usually in a latent, uncomplexed form and can be further induced by stresses. Conversely, tumours often express high levels of catalytically active HSP90, which is found in complexes with oncogenic client proteins [19]. HSP90 is overexpressed in breast tumours, lung cancer, leukaemia (i.e., myelodysplastic syndromes and acute myeloid leukaemia), and Hodgkin lymphoma [20], and thus it contributes to tumorigenicity and cancer cell resistance. HSP90 acts through both its antiapoptotic role and its chaperone function of stabilizing many kinases involved in cancer-cell signalling, including tyrosine kinases (i.e., FLT3 [21], JAK2 [22], v-Src [23], and serine/threonine kinases (AKT, Raf-1)). HSP90 also interacts with and stabilizes the receptor interacting kinase (RIP). Upon stimulation by Tumour Necrosis Factor alpha (TNF-*α*), RIP is recruited to the TNF receptor, thereby allowing the activation of Nuclear Factor-kappa B (NF-*κ*B), a key component of the inflammatory response. Following depletion of HSP90, RIP is degraded and activation of NF-*κ*B is prevented [24]. In this context, HSP90, by its stabilizing effect on the conformations of mutant proteins during transformation, favours the emergence of polymorphisms and mutations that support the evolution of resistant clones. Therefore, a rationale exists for targeting HSP90-dependent pathways in cancers and inflammatory diseases.

*The Chaperone HSP70*. The HSP70 family constitutes the most conserved and well-known class of HSPs. Among them, the inducible HSP70 (also called HSP72/HSPA1) and the constitutively expressed HSC70 (HSP73/HSPA8) are mainly localized in the cytosol, while others are located in the mitochondria (mtHSP70) or in the endoplasmic reticulum (GRP78/Bip). HSP70 cochaperones (HSP40, HSP110, CHIP, HOP, HIP, BAG-1, and BAG-3) modulate the chaperone activity of the protein through their binding to functional domains of HSP70 either the NH2-terminal ATP-binding domain (ABD) or the COOH-terminal peptide-binding domain (PBD).

HSP70 is a powerful antiapoptotic protein that inhibits both caspase-dependent (extrinsic and intrinsic pathways) and independent cell death [25, 26]. The important role of HSP70 was further demonstrated using mouse embryonic cells that lacked inducible HSP70 (encoded by HSP70.1 and HSP70.3), which display hypersensitivity to a wide range of lethal stimuli [27].

The high expression of HSP70 is associated with a poor prognosis and resistance to chemotherapeutic drugs in many cancers such as breast, endometrial, or gastric cancer [28, 29]. In Bcr-abl leukaemia cells, the expression of the protein HSP70 is also elevated and requires the GATA-response element in the HSP70 promoter [30]. Recently, Gallardo and colleagues identified a role for HSP70 in the proliferation and survival of the erythroid lineage in PV. In an* ex vivo* model, inhibition of HSP70 expression led to the dose-dependent inhibition of cell growth and burst formation unit erythroid (BFU-E) in PV and ET. This effect was associated with increased apoptosis of the erythroid lineage and decreased phospho-JAK2 signalling [31]. HSP70 might contribute to cell proliferation through the regulation of STAT signalling. Overexpression of HSP70 has been shown to upregulate STAT5 levels and activity, thus allowing the expression of the antiapoptotic protein Bcl-xl [32].

## 2. Focus on Emerging Drugs as HSP Inhibitors

### 2.1. HSP90 Inhibitors

The HSP90 chaperone family has a unique pocket in their N-terminal region, which binds ATP and ADP. This domain is crucial for the control of conformation and activity. Most competitive inhibitors of HSP90 bind to this “pocket” and block interaction with its client proteins.

There are two major classes of active molecules: natural HSP90 inhibitors and their derivatives, as well as synthetic inhibitors. In this review, we focus on HSP90 inhibitors, which have been proved to be useful for patients with haematological malignancies.

Most of the natural HSP90 inhibitor derivatives come from Geldanamycin and Radicicol [33]. Geldanamycin is an ansamycin-derivative benzoquinone that binds to the ATP binding “pocket” of HSP90 with higher affinity than natural nucleotides. This compound was discovered in 1970 [34]. It was first identified for its antibiotic and antitumoural potential in leukaemia (L1210) and nasopharynx KB cell lines [34]. A preclinical study subsequently revealed the hepatotoxicity of this inhibitor, thereby limiting its application [35]. Many derivatives have been reported to have less severe hepatotoxic effects and demonstrate potent anticancer activity at nontoxic doses, as is the case for 17-allylamino-17-demethoxygeldanamycin (tanespimycin, 17-AAG) and 17-[2-(dimethylamino) ethyl] amino-17-demethoxygeldanamycin (alvespimycin, 17-DMAG) [36]. 17-DMAG, which has better bioavailability and water solubility, went through a phase I evaluation [37] and 17-AAG with an improved formulation (DMSO-free) is in a phase III clinical trial.

Similarly to Geldanamycin, Radicicol is a macrocyclic antifungal antibiotic [38] that binds to the N-terminal domain of HSP90 [39] and destabilizes HSP90 client proteins. While* in vitro* studies have shown the efficacy of Radicicol, it was found unstable* in vivo*. Derivatives of Radicicol like VER-52296 (NVP-AUY922) were thus developed and entered in clinical trials in 2007 [40].

In parallel with natural HSP90 inhibitor derivatives, novel synthetic inhibitors have been developed, including purine-based compounds. The first class of such scaffolds was the PU series, such as PU-H71 and PU-DZ8, which share biological activity with Geldanamycin and Radicicol [33]. The PU series have a higher affinity for HSP90 than does ADP and are more water soluble and specific [41]. One of them, PU-H71, has been shown to induce tumour regression in a xenograft model of triple-negative breast cancers [42] and is currently in clinical trials. Another family of chemical compounds, not yet disclosed, has been produced by Serenex using a chemoproteomics technology platform. Among this family, SNX-7081, a small oral molecule, is more potent than 17-AAG in Chronic Lymphocytic Leukaemia [43] and SNX-5422 has entered a phase I trial [43] (Pfizer Inc., New York, NY, USA) [44, 45].

### 2.2. HSP70 Inhibitors

Members of the HSP70 family comprise three major domains: an N-terminal domain, which binds ATP, a substrate-binding domain, and a C-terminal domain, which also acts as a substrate-binding domain. Inhibitors of HSP70 could be classified in two groups according to the targeted domains, ABD or PBD.

Different inhibitors targeting the PBD have been developed. The first inhibitor was derived from the apoptosis inducing factor (AIF) protein, named ADD70 (AIF derived decoy for HSP70) [27]. It prevents caspase-independent cell death through its association with HSP70 [46]. Other small chemical inhibitors of HSP70, like 2-phenylethynesulfonamide (PES), which is also called pifithrin-*μ*, induce an apoptotic caspase-dependent cell death [47].

Other strategies to inhibit HSP70 are based on molecules that target the ABD. The first inhibitor tested was VER-155008. This is an adenosine-derived inhibitor that inhibits cell proliferation and induces apoptosis by targeting the ABD of both the inducible and constitutive (HSC70) form of HSP70 [48]. Furthermore, combined with small HSP90 inhibitors, VER-155008 displays a potentiator effect in colon cancer cells [48].

In order to protect the activity of constitutive HSC70, peptide inhibitors selectively directed against the inducible form of HSP70 have been designed. By screening, our group has selected peptide aptamers with (i) high affinity for the ABD of HSP70 and (ii) a strong inhibitory capacity on the chaperone activity of HSP70* in vitro*. One of these aptamers, named A17, has also demonstrated a strong antitumoural potential in* vivo* [49].

Designing molecules that directly target HSP70 protein is not the only strategy. Other molecules, like KNK437 (N-formyl-3,4-methylenedioxy-benzylidene-gamma-butyrolactam), are able to inhibit the expression of inducible HSPs. In particular, KNK434 has been shown to prevent the induction of HSP70 following treatment with HSP90 inhibitors [50].

## 3. HSP90 and HSP70: Role in the Inflammatory Process

### 3.1. HSP90 and Inflammation

As previously mentioned, HSP90 functions as part of a multichaperone complex via its association with cochaperones (e.g., p23 and HSP70) and client proteins. Inhibition of HSP90 by inhibitors such as benzoquinone ansamycins leads to both upregulation of HSP expression (especially HSP70 and HSP27) and degradation of various client proteins via proteasomal degradation [51]. Several key regulators of signalling pathways that play critical roles in mediating inflammatory and immune responses are clients of HSP90. These include JAK/Signal Transducer and Activator of Transcription (STATs) (JAK2), Toll Like-Receptor- (TLR-) 4 (e.g., TGF-*β*-activated kinase- (TAK-) 1 and RIP kinase), and NF-*κ*B (Inhibitor of I*κ*B Kinase (IKK)) signalling pathways [52].

Although the main application of HSP90 inhibitors is related to cancer therapy, these drugs are potent inhibitors of certain proinflammatory mediators in different cell types [50, 51]. The use of HSP90 inhibitors causes the dissociation of the IKK complex and thus prevents NF-*κ*B activation [53]. In a number of* in vitro* models, Geldanamycin has inhibited TNF-*α*-mediated IKK and NF-*κ*B activation [24, 50, 54]. In cultured human respiratory epithelium, Geldanamycin also inhibits TNF-alpha-mediated IL-8 gene expression [54]. SNX-7081 (by Serenex), a potent inhibitor of HSP90, increases HSP70 levels, and prevents NF-*κ*B nuclear translocation, and cytokine and nitric oxide (NO) production following the stimulation of Jurkat cells by TNFalpha, Interleukin(IL)1-beta or LPS [55]. Data show that HSP90 immunostaining is increased in inflammatory regions of human atherosclerotic plaque. Atherosclerotic plaque of mice treated with either 17-AAG or 17-DMAG showed reduced activation of the transcription factors STATs and NF-*κ*B and chemokine expression induced by proinflammatory cytokines [56]. 17-AAG was also shown to attenuate the inflammatory response in autoimmune encephalomyelitis and in severe sepsis [55, 57]. More recently, Yun et al. showed that a synthetic HSP90 inhibitor, EC144, is potent and selective and is efficacious in an inflammatory mouse model of endotoxic shock. EC144 is able to prevent LPS-mediated TLR4 signalling, thus decreasing proinflammatory cytokines, such as TNF-*α* and IL-6 [58].

### 3.2. HSP70 and Inflammation

Inflammation occurs in response to various cellular stresses including infection or heat shock. During infection, the level of HSP70 is increased and confers cytoprotection [59] via the inhibition of inflammatory signalling pathways, including the NF-*κ*B pathway [60].

HSP70 proteins are implicated in the regulation of the immune responses and modulate inflammation through several mechanisms. Of note, extracellular HSP70 is released from intact cells via an active nonclassical secretory pathway. HSP70 could be secreted associated with exosomes or by an endolysosomal dependent pathway [61, 62]. Extracellular HSP70 was first shown to enhance the cross-presentation of the HSP-bound peptide by MHC-I on dendritic cells (DCs). HSP70s, by associating with peptides, form an HSP-peptide complex that binds to cell surface receptors, such as CD91 and Lox-1, and is further internalized by endocytosis [63]. Extracellular HSP70 functions as a “danger signal” [64], since, compared with serum from healthy individuals, serum from patients with autoimmune diseases, patients with trauma, and children with septic shock shows high concentrations of HSP70 [65–67]. As a mediator of inflammation, exogenous HSP70 is able to activate cell surface receptors on immune cells such as TLR2 and TLR4 [68], thus leading to NF-*κ*B activation, and TNF-*α*, IL1-*β*, and IL-6 production [69]. As well as activating the NF-*κ*B pathway, downstream signalling induced by HSP70 elicits a rapid calcium flux, and the production of inflammatory cytokines and chemokines by monocytes and DCs [69].

## 4. HSP90 as a Therapeutic Target in MPN and the Potential Implication of HSP70

Malignant cells have more unfolded proteins than do normal cells, which lead to higher chaperone expression. In transformed myeloid cells, oncogenic transducer molecules have been shown to depend more on HSP90 chaperoning than is the case in untransformed cells [70]. Among the client proteins that interact with and are stabilized by HSP90, there are several transducer molecules, such as kinases like BCR-ABL [71, 72], FLT-3 [21], and JAK2 [22]. In myeloproliferative neoplasms, one promising therapy is based on using HSP90 as a therapeutic target to destabilize its oncogenic partners and induce their proteolytic degradation. Different specific inhibitors of HSP90 (i.e., geldanamycin derivatives, resorcinol derivatives, purine analogues, and other synthetic inhibitors) are currently used as anticancer drugs and display potent activity in clinical trials in patients with solid tumours and haematological malignancies [42, 68].

It was first established that targeting HSP90 in JAK2-mutant cells lines by using 17-AAG reduced JAK2 levels and inhibited JAK2 signalling pathway activation [73]. This study showed a decrease in the expression of oncoprotein AKT and in the phosphorylated form of STAT5 and JAK2. In addition to this direct inhibition strategy, the authors combined JAK2 inhibitors with 17-AAG and observed no synergic effect. However, a more detailed study that examined the effects of PU-H71 [74] showed promising results in preclinical models of solid cancers [42] or haematological cancers [75]. In addition, the toxic side effects of PU-H71 on nonhaematopoietic cells were lower than those associated with geldanamycin derivatives, such as 17-AAG or 17-DMAG [74]. In this work, PU-H71 demonstrated interesting effects* in vitro* in JAK2-dependent cell lines,* in vivo* in murine models of PV and ET, and in samples from patients with primary MPN [74]. Moreover, PU-H71, which is able to induce degradation of client proteins of HSP90 including JAK2, inhibits proliferation and attenuates JAK-STAT pathways in JAK2V617F-positive or MPLW515L-positive cell lines. Following these results, the authors observed additive effects of a combined treatment using PU-H71 and a JAK2 inhibitor (TG101348), suggesting that using inhibitors of HSP90 alone or in combination might be an interesting therapeutic approach.

In light of these results, other inhibitors of HSP90 were tested. Among these molecules, a nongeldanamycin analogue (NVP-AUY922, AUY922), already known to inhibit the chaperone association of HSP90 with its client proteins [72, 75], was investigated in the context of a preclinical MPN study [70]. This study found a synergic effect of AUY922 and a JAK2 inhibitor (TG101209) in JAK2V617F cells lines. In addition, it has been shown that genetic resistance to enzymatic inhibitors of JAK2 is overcome by HSP90 inhibition [76]. This combination was also evaluated on CD34+ cells harvested from the peripheral blood of patients suffering from myelofibrosis. In these primary cells, the combined treatment was more effective than each agent alone and caused a greater decrease in JAK2 and AKT levels in addition to a decrease in the phosphorylated forms of STAT3 and STAT5 without affecting their expression. More recently, it was also observed that the effect of JAK inhibitors and especially the dual JAK1/JAK2 inhibitor INCB18424 (ruxolitinib, JAKAVI) was enhanced by PU-H71 in a murine model of myelofibrosis [77]. Nevertheless, HSP90 inhibitors have been reported to increase the expression of other HSPs including HSP70, which provides a selective survival advantage in tumour cells.

The implication of HSP70 in MPN has not been investigated; however, several in-depth studies have explored the implication of HSP70 in other types of haematological malignancies including chronic [78, 79] or acute myeloid leukaemia [80]. Recently, proteomic analysis of samples from PV and ET patients revealed that HSP70 is overexpressed in PV samples but not in ET samples. Additionally, JAK2V617F cells from patients with PV were sensitized by siRNA HSP70 interference assay or treatment with an inhibitor of HSP70 (KNK437). Their results supported the hypothesis that HSP70 is implicated in the pathogenesis of PV and suggested that HSP70 could constitute a new molecular target [31]. Furthermore, the association of ruxolitinib with the HSP70 inhibitor, KNK437, showed a synergic effect in both JAK2 V617F cells lines and in culture colonies of PV [81]. These data confirm that HSP70 must participate in the pathogenesis of PV and seems to be a promising therapeutic target in MPN.

## 5. Concluding Remarks

**Figure 1:**
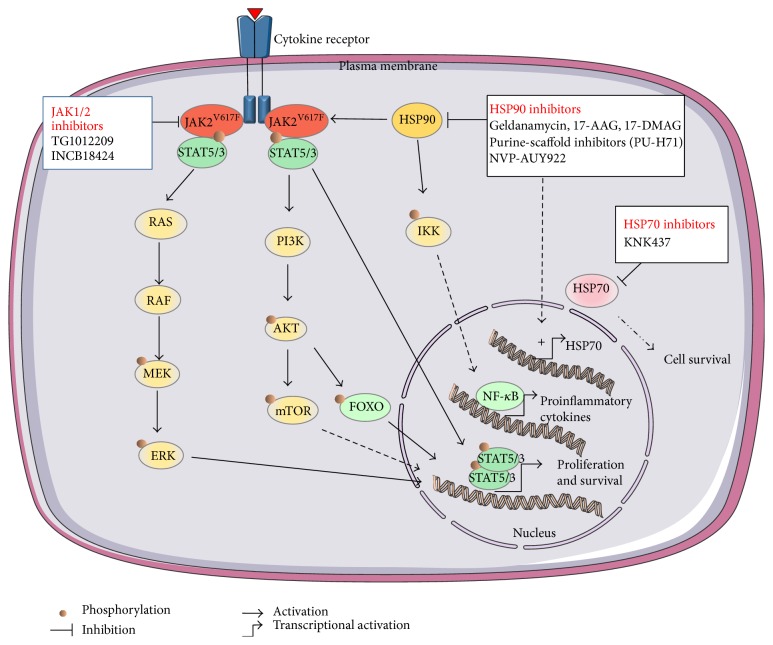
Schematic representation of the potential effects of HSP inhibitors on the main activated pathways in myeloproliferative neoplasms. PI3K (Phosphatidyl-Inositol-3 Kinase), STAT (Signal Transducers and Activators of Transcription), ERK (extracellular signal-regulated kinases), MEK (mitogen-activated protein kinase kinase), HSP (heat shock protein), mTOR (mammalian target of rapamycin), FOXO (Forkhead transcription factors), NF-*κ*B (Nuclear Factor-kappa B), and IKK (Inhibitor of I*κ*B Kinase).

Inhibition of HSP90 as a therapeutic approach was first evaluated in the context of cancer treatments [19, 77]. However, growing evidence indicates that HSP90 inhibitors could be beneficial for both MPN and inflammatory reactions. Moreover, and given the substantial increase in HSP70 induced by HSP90 inhibitors, a combination with HSP70 inhibitors might be an interesting alternative to optimise the treatment [74, 75] (Figure 1).
